# Contrasting effects of DNA demethylation on cancer-germline gene expression in breast cancer and leukemia cells

**DOI:** 10.1371/journal.pone.0339460

**Published:** 2025-12-18

**Authors:** Mikhlid H. Almutairi, Turki M. Alrubie

**Affiliations:** 1 Department of Zoology, College of Science, King Saud University, Riyadh, Saudi Arabia; 2 National Livestock and Fisheries Development Program, Riyadh, Saudi Arabia; Qatar Biomedical Research Institute, QATAR

## Abstract

Human germline gene expression is normally constrained to the germ cells, responsible for the production of sperm and oocytes. Cancer-germline (CG) genes, a subset of germline genes involved in testis development, are frequently aberrantly activated in cancer cells. The present study investigates the broader hypothesis that epigenetic modifications, specifically DNA methylation, can modulate the expression profiles of several CG genes in cancer and germ cells. Breast cancer (BC), normal breast (NB), and chronic myelogenous leukemia (CML) cell lines were treated with the DNA methyltransferase inhibitor (DNMTi) 5-aza-2’-deoxycytidine for three days. The effects of this treatment on the transcriptional activation of CG genes (*SYCP1*, *ADAD1*, *SYCE1*, *PRSS54*, *DMRTC2*, and *TEX101*) were then evaluated. We comprehensively analyzed differential methylation, survival analysis (Kaplan-Meier), correlation (Spearman’s), and pathway enrichment analysis (GO/KEGG) of CG genes (SYCP1, ADAD1, SYCE1, PRSS54, DMRTC2, and TEX101) in BC and leukemia. Treatment with 5-aza-2’-deoxycytidine upregulated CG genes in BC cells but downregulated them in leukemia cells, highlighting tissue-specific epigenetic responses. Differential methylation analysis revealed cancer-specific patterns: ADAD1 was hypermethylated in both malignancies, while PRSS54 was hypomethylated in leukemia. Survival analysis linked SYCE1 and PRSS54 to prolonged survival in BC, whereas TEX101 and SYCP1 correlated with poorer outcomes. Functional enrichment identified ADAD1 and SYCP1 as key players in BC and leukemia pathways, respectively. Meta-analysis validated SYCP1 as a robust biomarker with consistent effect sizes across datasets. Methylation-expression correlations were stronger in tumors, with SYCE1 and DMRTC2 showing inverse relationships in leukemia. These findings demonstrate that the expression of a subset of CG genes is responsive to modulation by hypomethylating drugs in a tissue-specific manner, highlighting their promise as candidates for future investigation in cancer immunotherapy.

## Introduction

Germline-specific genes are typically activated in cancer cells and contribute to cancer progression; these genes are known as cancer-germline genes (CGs). The CG antigens hold significant promise for cancer immunotherapy [1,2]. While their expression in healthy adult tissues is primarily restricted to testicular germ cells, they are found in a wide range of cancers [3,4]. The restricted expression pattern, along with their ability to stimulate an immune response, makes CG antigens attractive candidates for targeted therapies [3,5].

DNA methylation, a key epigenetic modification, plays a critical role in maintaining CG gene silencing in somatic tissues [6]. In cancer, global hypomethylation is a well-established hallmark that contributes to genomic instability and aberrant gene expression [7]. While this often leads to CG gene reactivation, the relationship between DNA demethylation and CG gene expression is not uniform across all genes or cancer types [8]. Previous studies have demonstrated that treatment with DNA methyltransferase inhibitors (DNMTis), such as 5-aza-2’-deoxycytidine (decitabine; 5-aza-CdR), can induce CG gene expression in some, but not all, cancer cell lines [9]. This variability suggests that additional layers of regulation, including histone modifications, transcription factor availability, and chromatin structure, influence CG gene reactivation potential [10].

DNA methyltransferases (DNMTs) are enzymes responsible for attaching a methyl group (CH3) to the 5’ position of cytosine residues within CpG (cytosine-phosphate-guanine) dinucleotides [11]. An increase in methylation within the promoter region, known as promoter hypermethylation, often results in gene silencing by methylating CpG islands. In contrast, promoter hypomethylation involves decreased methylation levels, which can lead to the activation of previously silenced genes in cancer cells [12]. The reversible nature of epigenetic modifications has driven the development of epigenetic therapies aimed at evaluating their potential as treatments for various cancers [13]. For example, epigenetic drugs, such as DNA methyltransferase inhibitors, have been demonstrated to upregulate the expression of CG genes within tumors [14]. This increased expression may enable the immune system to recognize these genes as foreign, offering promising avenues for cancer immunotherapy strategies [14,15].

Breast cancer and leukemia represent two malignancies with distinct biological characteristics and epigenetic landscapes [16]. Breast cancers frequently exhibit CG gene expression, particularly in more aggressive subtypes such as triple-negative breast cancer (TNBC), and this has been linked to poor prognosis and therapeutic resistance [17]. In contrast, leukemias exhibit more variable patterns of CG gene expression, with some genes consistently activated while others remain silent, despite global hypomethylation [18]. These differences may reflect tissue-specific variations in the epigenetic machinery or distinct evolutionary pressures during tumor development [19].

The expression of numerous CG antigens is regulated by the extent of DNA methylation in their promoter regions. Reduced methylation is associated with increased gene expression. Consequently, the upregulation of many CG antigen genes in various cancers is often attributed to genome hypomethylation that occurs during carcinogenesis [3,20,21]. Importantly, treatment with DNA methyltransferase inhibitors (DNMTi) can increase CG gene expression in cancer cells, particularly for X-linked CG genes [X-CT], which comprise approximately half of all identified CG genes [5]. For instance, Almatrafi et al. (2014) demonstrated that several CG genes, previously silenced through CpG island hypermethylation, could be activated by DNA hypomethylating agents [5]. Other research has indicated a similar trend for various CG genes [21–23]. Colemon et al. (2020) found a distinct correlation between MAGE-A gene methylation and expression levels in several cancerous tissues [22]. Likewise, studies have demonstrated that promoter hypomethylation leads to increased expression of the NY-ESO-1 gene in both non-small cell lung cancer [24] and acute myeloid leukemia [25]. Therefore, DNMTs possess the capacity to upregulate CG antigen expression and potentially improve the efficacy of immunotherapy [3,5,20]. This suggests that it would be advantageous to include a DNMTi in clinical cancer immunotherapy protocols

An in-silico approach identified a new group of CG genes, and their expression has been experimentally confirmed in cancer cell lines [4,23,26]. Recent studies have identified high expression levels of several of these genes in tumor samples from Saudi patients diagnosed with colon cancer (CC), compared to paired normal colon (NC) tissues [3,27]. The majority of these genes are found on autosomal chromosomes. Furthermore, another study found no detectable expression of the CG genes in either the CC patient samples or the NC tissues, leading to their classification as testis-specific (restricted) genes, as their expression was restricted to normal testis tissue [1].

This study hypothesized that epigenetic processes, including DNA methylation, influence the expression of identified CG or testis-specific genes, such as SYCP1, ADAD1, SYCE1, PRSS54, DMRTC2, and TEX101, in breast cancer (BC), normal breast (NB), and chronic myelogenous leukemia (CML) cell lines. The current research explored the impact of epigenetic alteration, specifically the introduction of a DNMT inhibitor (DNMTi), on the transcriptional activation of known CG genes. The findings of this investigation contribute to a more comprehensive understanding of the regulatory processes controlling these genes in BC and CML tissues.

## Materials and methods

### Human BC, CML, and NB cell line sources and cultures

The human BC cell line MCF-7, the CML cell line K562, and the NB cell line MCF-10A were used in this study. MCF-7 and K562 cells were obtained from the American Type Culture Collection (ATCC; Manassas, VA, USA). Dr. Bader Almutairi, King Saud University, provided MCF-10A. MCF-7 and K562 cells were cultured in Dulbecco’s Modified Eagle Medium (DMEM; Thermo Fisher Scientific; 61965026) supplemented with 10% fetal bovine serum (FBS; Thermo Fisher Scientific; A3160801). MCF-10A cells were cultured in DMEM supplemented with 10% FBS, 2 mM glutamine, 100 IU/mL penicillin, and 100 μg/mL streptomycin (all growth factors sourced from Sigma, St. Louis, MO, USA). All cell lines were maintained in a humidified incubator at 37°C with 5% CO_2_.

### 5-Aza-2′-CdR treatment

MCF-7, K562, and MCF-10A cells were treated with 5-aza-2′-CdR (Sigma; A3656) or dimethyl sulfoxide (DMSO) as a vehicle control. 5-Aza-2′-CdR was dissolved in DMSO to create a stock solution, which was then diluted to a final concentration of 10 μM in culture medium. The culture medium was changed daily during the treatment, and 5-aza-2′-deoxycytidine was readministered 24 hours after the initial dose to maintain its effectiveness. Cells were exposed to 10 μM 5-aza-2′-CdR or DMSO for 3 days (72 hours). The treatment duration and concentration were based on previously published findings [3,5,20].

### Total RNA isolation and cDNA synthesis from BC, CML, and NB cell cultures

Total RNA was extracted from around 5 x 10^6^ grown cells using the AllPrep DNA/RNA Mini Kit according to the manufacturer’s instructions (Qiagen, Hilden, Germany; Cat. No. 80204). A NanoDrop 8000 spectrophotometer was used to measure the concentration, purity, and quality of RNA. The High-Capacity cDNA Reverse Transcription Kit (Applied Biosystems, Waltham, MA, USA; Cat. No. 4368814) was used to reverse transcribe two micrograms (2 μg) of total RNA into cDNA following the manufacturer’s instructions. The resultant cDNA was kept at −20°C until it was needed again after being diluted 1:11 in nuclease-free water.

### Primer design and validation for qRT-PCR assay

Approximately 175 bp amplicons were intended to be amplified by the primers used for qRT-PCR. Manually constructed primer sequences (50–55% GC content, 20 nucleotides long) were designed to reduce internal secondary structure and the possibility of primer-dimer formation. Minimal 3’ complementarity and comparable melting temperatures were used in the construction of the forward and reverse primers. Using the appropriate genome database and a BLAST search, primer specificity was verified. Primer sequences are listed in Table 1.

**Table 1:** Sequences of the primers used in the qRT-PCR.

Official gene symbol	Gene ID	Primer sequences (Forward = F; Reverse = R)	Product length (bp)	Gene location
*GAPDH*	2597	**F:** GGGAAGCTTGTCATCAATGG **R:** GAGATGATGACCCTTTTGGC	173	Chromosome 12 (12p13.31)
*ACTB*	60	**F:** AGGAGAAGCTGTGCTACGTC **R:** GGAAGGAAGGCTGGAAGAGT	162	Chromosome 7 (7p22.1)
*SYCP1*	6847	**F:** TTCAGAGGGATTGAGCAGAG **R:** CCTGAATGGCTTTTCGCTGT	155	Chromosome 1 (1p13.2)
*ADAD1*	132612	**F:** ATGGCATCCAAGGTTACGCA **R:** AAACTGGTGCAAGGCTGACA	168	Chromosome 4 (4q27)
*SYCE1*	93426	**F:** GAGGTCCTGATTAACCGGAT **R:** CTCAAGATCTCCTTCAGGTG	149	Chromosome 10 (10q26.3)
*PRSS54*	221191	**F:** GGAAACCAAGACTGCCTGCT **R:** GCTGTAGTCTTCCACCTTGG	151	Chromosome 16 (16q21)
*DMRTC2*	63946	**F:** CAAATGTGTCCTCATCCTGG **R:** TCTGAAGTGGTTGGGAGCTT	136	Chromosome 19 (19q13.2)
*TEX101*	83639	**F:** TCTGTCCATGACTGTGGAAG **R:** GCCAAAATGGCTGTCTCAGT	138	Chromosome 19 (19q13.31)

### Set up for qRT-PCR

Following the manufacturer’s instructions, qRT-PCR was carried out using the iTaq Universal SYBR Green Supermix (Bio-Rad, Hercules, CA, USA; Cat. No. 1725120). 5 μL of SYBR Green Supermix, two μL of diluted cDNA template (equal to 180 ng of reverse transcribed RNA), 0.25 μL of forward primer, 0.25 μL of reverse primer (both at 10 pmol/μL), and 2.5 μL of nuclease-free water were used in each 10 μL reaction. A QuantStudio 7 Flex Real-Time PCR System (Applied Biosystems, Waltham, MA, USA) was used to conduct the reactions in triplicate. The cycling conditions were as follows: initial denaturation at 95°C for 30 s, 40 cycles of denaturation at 95°C for 30 s, annealing at 58°C for 30 s, and extension at 72°C for 30 s. A melt curve analysis was performed immediately after amplification to confirm the specificity of the qRT-PCR product. *GAPDH* and *ACTB* were used as the reference genes for normalization.

### Statistical analysis

For every gene, three independent qRT-PCR tests were conducted. The mean ± standard deviation (SD) was used to display the data. GraphPad Prism version 5 was used to establish statistical significance (GraphPad Software, San Diego, CA, USA). P values were deemed statistically significant if they were less than 0.05. Levels of significance are shown as follows: *P ≤ 0.05; ** P = 0.01; *** P = 0.001; **** P = 0.0001.

### *In silico* analysis

#### Data acquisition and preprocessing.

To investigate the methylation profiles of SYCP1, ADAD1, SYCE1, PRSS54, DMRTC2, and TEX101 in BC and leukemia, we utilized publicly available datasets from The Cancer Genome Atlas (TCGA) [28]. DNA methylation data (Illumina Infinium HumanMethylation450K and EPIC arrays) were retrieved for breast invasive carcinoma (TCGA-BRCA) and acute myeloid leukemia (TCGA-LAML) cohorts. Beta values (β), representing methylation levels (ranging from 0 = unmethylated to 1 = fully methylated), were extracted for each gene’s promoter and CpG island regions using the UCSC Xena browser and GDC Data Portal. Batch effects were corrected using ComBat (sva R package), and probes with detection p-values > 0.01 were excluded.

#### Differential methylation analysis.

We performed comparative methylation analysis between tumor and adjacent normal tissues (where available) or healthy controls. For BC, we compared TCGA-BRCA tumor samples (n = 1,098) against NB tissue (n = 114). For leukemia, TCGA-LAML tumor samples (n = 200) were analyzed against whole blood methylation data from healthy donors (GEO: GSE40279). Differential methylation was assessed using limma (R/Bioconductor), with a significance threshold of |Δβ| ≥ 0.2 and adjusted p-value (FDR) < 0.05. Genes with hypermethylation (Δβ > 0.2) in tumors were considered epigenetically silenced, while hypomethylation (Δβ < −0.2) indicated potential activation.

#### Survival and clinical correlation analysis.

To evaluate the prognostic significance of methylation changes, we conducted Kaplan-Meier survival analysis using TCGA clinical data. Patients were stratified into high- and low-methylation groups based on median β-values for each gene. Log-rank tests determined differences in overall survival (OS) and disease-free survival (DFS). Additionally, we correlated methylation levels with clinicopathological features (e.g., tumor stage, subtype, and metastasis) using Spearman’s rank correlation.

#### Functional enrichment and pathway analysis.

To explore the biological implications of methylation alterations, we performed Gene Ontology (GO) and Kyoto Encyclopedia of Genes and Genomes (KEGG) pathway enrichment analyses on co-methylated genes using clusterProfiler (R). Genes showing significant methylation changes in SYCP1, ADAD1, SYCE1, PRSS54, DMRTC2, and TEX101 were cross-referenced with STRING-DB to identify protein-protein interaction networks.

#### Validation using independent cohorts.

To confirm our findings, we analyzed independent methylation datasets from GEO (e.g., GSE69914 for BC, GSE63409 for leukemia). Meta-analysis was performed using random-effects models (METAL software) to assess consistency across studies.

#### Integration with transcriptomic data.

To determine if methylation changes corresponded with gene expression, we extracted RNA-seq data (TCGA-BRCA and TCGA-LAML) and calculated Pearson correlations between methylation β-values and gene expression (log2 [FPKM+1]). Genes with hypermethylation and downregulation (negative correlation) were classified as epigenetically repressed, while hypomethylation and upregulation (positive correlation) suggested epigenetic activation.

### Public ChIP–seq cross-check of histone marks at CG-gene loci

To address the potential mechanisms underlying the contrasting responses to DNMT inhibition, we analyzed publicly available histone modification ChIP-seq data from the ENCODE Project and Roadmap Epigenomics Consortium. We specifically interrogated the promoter regions (defined as ±2 kb from the canonical transcription start site) of SYCP1, DMRTC2, and TEX101 for the presence of activating (H3K27ac, H3K4me3) and repressive (H3K27me3) marks in relevant cell models. This analysis utilized specific ENCODE accessions for MCF-7 (e.g., ENCSR000EWR for H3K27ac) and K562 (e.g., ENCSR000AKU for H3K4me3), supplemented with available data for MCF-10A (e.g., GEO GSM2258705). Qualitative inspection of the chromatin landscape revealed a lineage-specific pattern: promoters in K562 cells lacked strong active marks and were enriched for the repressive Polycomb-associated mark H3K27me3, whereas the same regions in MCF-7 and MCF-10A epithelial cells exhibited a permissive state characterized by detectable H3K4me3 and reduced H3K27me3. These distinct chromatin environments provide a compelling epigenetic context for the observed differential gene expression responses to 5-aza-CdR treatment.

## Results

All X-linked CG genes are typically silenced by DNA hypermethylation of their regulatory sequences, and this silencing can be reversed by the hypomethylating agent 5-aza-CdR [4,20,29]. Given the potential clinical utility of upregulating immunogenic CG antigens, this study investigated whether identified autosomal CG genes and testis-specific genes [1,30] are also subject to silencing via a similar DNA hypermethylation mechanism.

### 5-aza-CdR induces expression of autosomal CG genes in MCF-7 and MCF-10A cell lines

**Fig 1:**
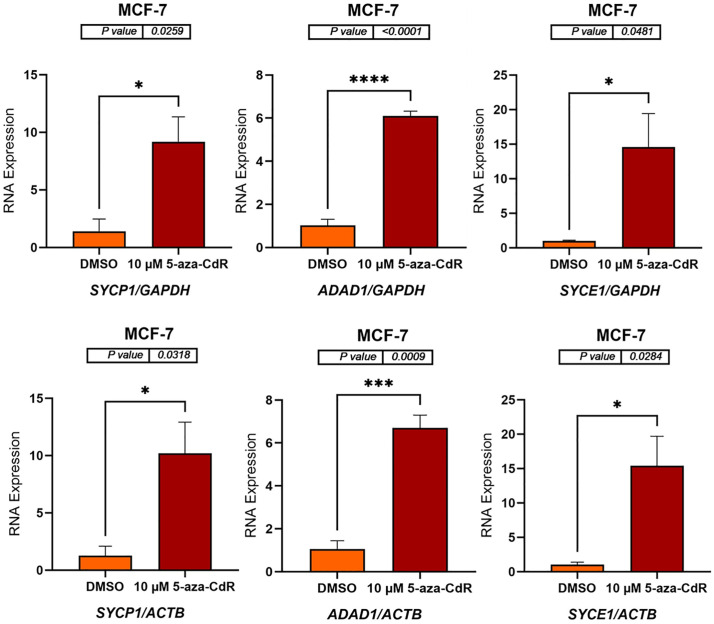
qRT-PCR analysis of *SYCP1*, *ADAD1*, and *SYCE1* expression in MCF-7 cells treated with either DMSO (control) or 10 μM 5-aza-CdR for three days. The bar charts display the relative RNA expression levels of *SYCP1*, *ADAD1*, and *SYCE1* before and after 5-aza-CdR treatment. Gene expression was normalized to either *GAPDH* (upper panels) or *ACTB* (lower panels), as indicated by the x-axis labels. The standard error of the mean for three replicates of each gene is shown by error bars. P-values are displayed above the bar charts, and asterisks indicate statistical significance between treatment groups (*P ≤ 0.05, ***P ≤ 0.001, ****P ≤ 0.0001). The data indicate that 5-aza-CdR treatment significantly increases the expression of all three genes in MCF-7 cells.

**Fig 2:**
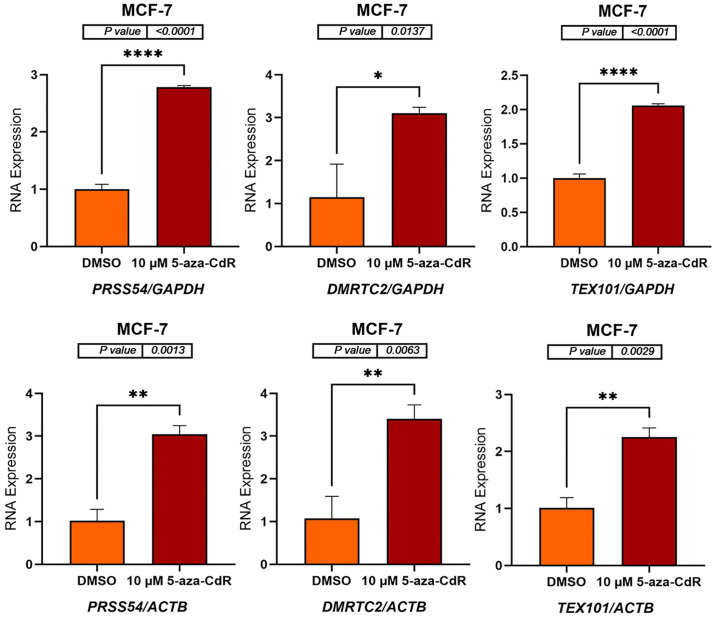
qRT-PCR analysis of *PRSS54*, *DMRTC2*, and *TEX101* expression in MCF-7 cells treated with either DMSO (control) or 10 μM 5-aza-CdR for three days. The bar charts display the relative RNA expression levels of *PRSS54*, *DMRTC2*, and *TEX101* before and after 5-aza-CdR treatment. Gene expression was normalized to either *GAPDH* (upper panels) and *ACTB* (lower panels), as shown on the x-axis labels. The standard error of the mean for three replicates of each gene is shown by error bars. P-values are displayed above the bar charts, and asterisks indicate statistical significance between treatment groups (*P ≤ 0.05, **P ≤ 0.01, ****P ≤ 0.0001). The data indicate that 5-aza-CdR treatment significantly increases the expression of all three genes in MCF-7 cells.

**Fig 3:**
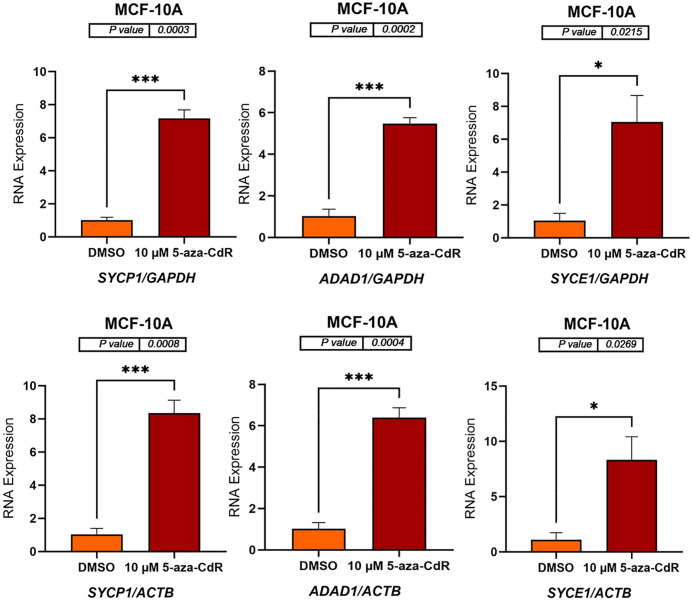
qRT-PCR analysis of *SYCP1*, *ADAD1*, and *SYCE1* expression in MCF-10A cells treated with either DMSO (control) or 10 μM 5-aza-CdR for three days. The bar charts display the relative RNA expression levels of *SYCP1*, *ADAD1*, and *SYCE1* before and after 5-aza-CdR treatment. Gene expression was normalized to either *GAPDH* (upper panels) and *ACTB* (lower panels), as shown on the x-axis labels. The standard error of the mean for three replicates of each gene is shown by error bars. P-values are displayed above the bar charts, and asterisks indicate statistical significance between treatment groups (*P ≤ 0.05, ***P ≤ 0.001). The data indicate that 5-aza-CdR treatment significantly increases the expression of all three genes in MCF-10A cells.

**Fig 4:**
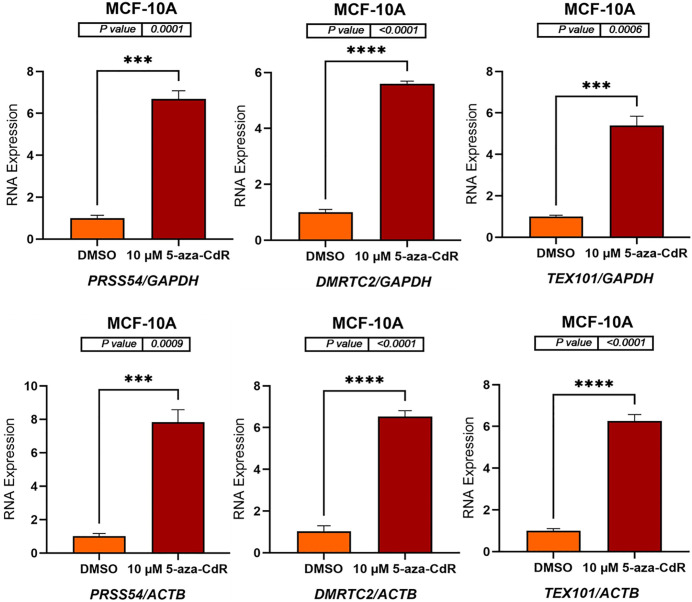
qRT-PCR analysis of *PRSS54*, *DMRTC2*, and *TEX101* expression in MCF-10A cells treated with either DMSO (control) or 10 μM 5-aza-CdR for three days. The bar charts display the relative RNA expression levels of *PRSS54*, *DMRTC2*, and *TEX101* before and after 5-aza-CdR treatment. Gene expression was normalized to either *GAPDH* (upper panels) and *ACTB* (lower panels), as shown on the x-axis labels. The standard error of the mean for three replicates of each gene is shown by error bars. P-values are displayed above the bar charts, and asterisks indicate statistical significance between treatment groups (***P ≤ 0.001, ****P ≤ 0.0001). The data indicate that 5-aza-CdR treatment significantly increases the expression of all three genes in MCF-10A cells.

To determine whether DNA methyltransferase inhibition could activate autosomal CG genes, MCF-7 and MCF-10A cell lines were treated with 10 μM 5-aza-CdR for three days. DMSO-treated cells served as a vehicle control due to the dissolution requirements of 5-aza-CdR. Following treatment, RNA was extracted, and complementary DNA (cDNA) was synthesized. *GAPDH* and *ACTB* were used as reference genes for normalization, as these genes are located on different autosomal chromosomes, allowing for the confirmation of results using independent normalization standards. The mRNA expression levels of six autosomal CG genes (*SYCP1, ADAD1, SYCE1, PRSS54, DMRTC2*, and *TEX101*) were subsequently measured by qRT-PCR. As shown in Figs 1 and 2 (MCF-7 cells) and Figs 3 and 4 (MCF-10A cells), 5-aza-CdR treatment results in a significant increase in the expression of all six genes compared to DMSO-treated controls, irrespective of whether normalization is performed against *GAPDH* or *ACTB*.

In MCF-7 cells, *SYCP1* expression increased by approximately 8-fold (p = 0.0259) and 10-fold (p = 0.0318) when normalized to *GAPDH* and *ACTB*, respectively. *ADAD1* expression showed a ~ 6-fold (p < 0.0001) and ~7-fold (p = 0.0009) increase relative to *GAPDH* and *ACTB*, respectively. *SYCE1* expression exhibited a ~ 14-fold (p = 0.0481) and ~15-fold (p = 0.0284) increase when normalized to *GAPDH* and *ACTB*, respectively (Fig. 1). *PRSS54* expression increased approximately 3-fold when normalized to *GAPDH* (p < 0.0001) and *ACTB* (p = 0.0013). *DMRTC2* expression also increased, showing roughly a 2.5-fold increase with *GAPDH* normalization (p = 0.0137) and approximately a 3-fold increase when normalized to *ACTB* (p = 0.0063). Similarly, *TEX101* expression showed a nearly 2-fold increase when normalized to *GAPDH* (p < 0.0001) and *ACTB* (p = 0.0029) (Fig 2). These findings indicate that 5-aza-CdR upregulates the expression of *SYCP1, ADAD1, SYCE1, PRSS54*, *DMRTC2*, and *TEX101* in MCF-7 cells.

In MCF-10A cells, treatment with 10 μM 5-aza-CdR significantly increased the expression of *SYCP1*, *ADAD1*, *SYCE1*, *PRSS54*, *DMRTC2*, and *TEX101*. *SYCP1* expression increased approximately 7-fold when normalized to *GAPDH* (p = 0.0003) and about 8-fold when normalized to *ACTB* (p = 0.0008). *ADAD1* expression showed a roughly 5-fold increase with *GAPDH* normalization (p = 0.0002) and approximately a 6-fold increase when normalized to *ACTB* (p = 0.0004). *SYCE1* expression exhibited approximately a 7-fold increase when normalized to *GAPDH* (p = 0.0215) and about an 8-fold increase when normalized to *ACTB* (p = 0.0269) (Fig 3). *PRSS54* expression increased approximately 6-fold when normalized to *GAPDH* (p = 0.0001) and about 7-fold when normalized to *ACTB* (p = 0.0009). *DMRTC2* expression showed roughly a 5-fold increase with *GAPDH* normalization (p < 0.0001) and approximately a 6-fold increase when normalized to *ACTB* (p < 0.0001). Similarly, *TEX101* expression exhibited around a 5-fold increase when normalized to *GAPDH* (p = 0.0006) and approximately a 6-fold increase when normalized to *ACTB* (p < 0.0001) (Fig 4). These findings indicate that 5-aza-CdR upregulates the expression of these genes in MCF-10A cells.

### 5-aza-CdR decreases the expression of autosomal CG genes in K562 cell line

**Fig 5:**
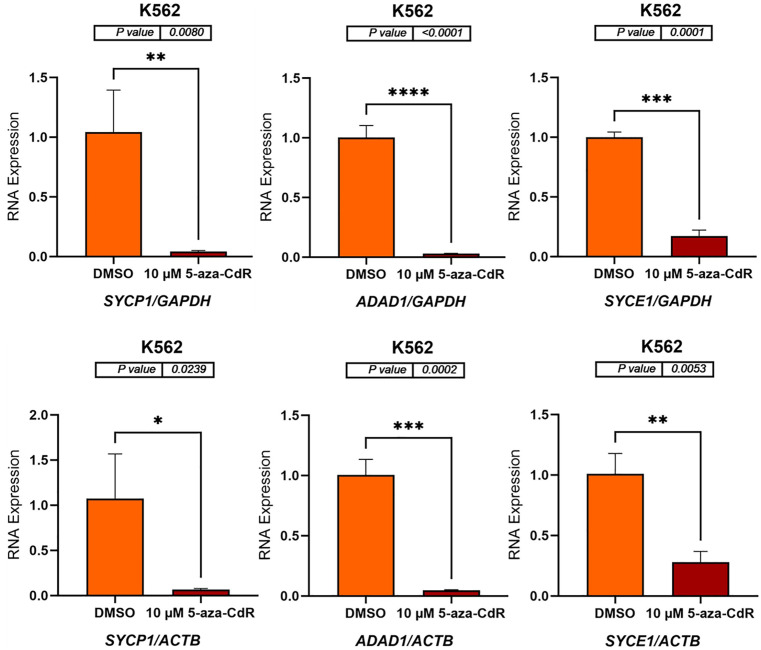
qRT-PCR analysis of *SYCP1*, *ADAD1*, and *SYCE1* expression in K562 cells treated with either DMSO (control) or 10 μM 5-aza-CdR for three days. The bar charts display the relative RNA expression levels of *SYCP1*, *ADAD1*, and *SYCE1* before and after 5-aza-CdR treatment. Gene expression was normalized to either *GAPDH* (upper panels) and *ACTB* (lower panels), as shown on the x-axis labels. The standard error of the mean for three replicates of each gene is shown by error bars. P-values are displayed above the bar charts, and asterisks indicate statistical significance between treatment groups (*P ≤ 0.05, **P ≤ 0.01, ***P ≤ 0.001, ****P ≤ 0.0001). The data indicate that 5-aza-CdR treatment significantly decreases the expression of all three genes in K562 cells.

**Fig 6:**
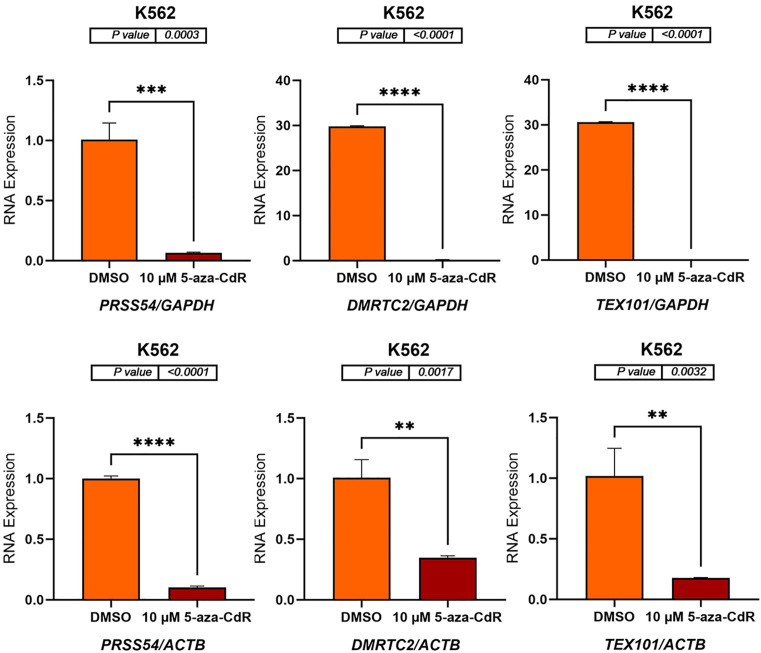
The bar charts display the relative RNA expression levels of *PRSS54*, *DMRTC2*, and *TEX101* before and after 5-aza-CdR treatment. Gene expression was normalized to either *GAPDH* (upper panels) and *ACTB* (lower panels), as shown on the x-axis labels. The standard error of the mean for three replicates of each gene is shown by error bars. P-values are displayed above the bar charts, and asterisks indicate statistical significance between treatment groups (**P ≤ 0.01, ***P ≤ 0.001, ****P ≤ 0.0001). The data indicate that 5-aza-CdR treatment significantly decreases the expression of all three genes in K562 cells.

In K562 cells, treatment with 10 μM 5-aza-CdR resulted in a significant decrease in the expression of *SYCP1*, *ADAD1*, *SYCE1*, *PRSS54*, *DMRTC2*, and *TEX101* (Figs 5 and 6). *SYCP1* expression decreased to approximately 0.1-fold when normalized to *GAPDH* (p = 0.0080) and *ACTB* (p = 0.0239). Similarly, *ADAD1* expression showed a roughly 0.1-fold decrease with *GAPDH* normalization (p < 0.0001) and *ACTB* normalization (p = 0.0002). *SYCE1* expression exhibited around a 0.2-fold decrease when normalized to *GAPDH* (p = 0.0001) and *ACTB* (p = 0.0053) (Fig 5). *PRSS54* expression decreased to approximately 0.1-fold when normalized to *GAPDH* (p = 0.0003) and *ACTB* (p < 0.0001). *DMRTC2* expression showed roughly a 0.01-fold decrease with *GAPDH* normalization (p < 0.0001) and approximately a 0.3-fold decrease when normalized to *ACTB* (p = 0.0017). Similarly, *TEX101* expression exhibited a decrease of approximately 0.01-fold when normalized to *GAPDH* (p < 0.0001) and a decrease of approximately 0.2-fold when normalized to *ACTB* (p = 0.0032) (Fig 6). These findings indicate that 5-aza-CdR downregulates the expression of these genes in K562 cells.

### Comparison of methylation levels between tumor and normal samples

**Fig 7:**
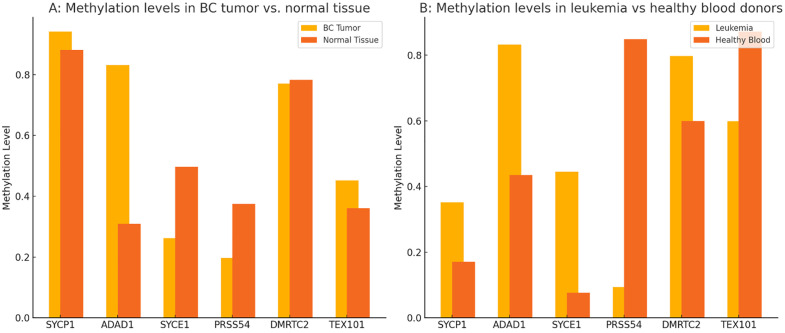
Differential DNA methylation patterns of CG genes (SYCP1, ADAD1, SYCE1, PRSS54, DMRTC2, and TEX101) in cancer. **(A)** Breast cancer (BC) tumors are compared to normal breast tissues. **(B)** Leukemia samples versus healthy blood donors.

In BC tissues compared to normal breast tissues (Fig 7A), SYCP1 showed a slight increase in methylation (~0.92 vs. ~ 0.87), while ADAD1 exhibited a substantial rise (~0.84 vs. ~ 0.31). In contrast, SYCE1 and PRSS54 displayed reduced methylation in BC tumor (~0.26 vs. ~ 0.52 and ~0.20 vs. ~ 0.37, respectively). DMRTC2 maintained similar methylation levels in both BC tumor and normal breast samples (~0.77 vs. ~ 0.78), whereas TEX101 was modestly higher in BC tissues (~0.46 vs. ~ 0.36). In leukemia versus healthy blood donors (Fig 7B), SYCP1, ADAD1, and SYCE1 were hypermethylated (~0.35 vs. ~ 0.17, ~ 0.84 vs. ~ 0.44, and ~0.45 vs. ~ 0.09, respectively), while PRSS54 was markedly hypomethylated (~0.08 vs. ~ 0.86). DMRTC2 showed elevated methylation in leukemia (~0.80 vs. ~ 0.60), whereas TEX101 was higher in healthy blood (~0.85 vs. ~ 0.60). These results highlight distinct methylation patterns between solid and hematological malignancies, with both hypo- and hypermethylation events contributing to differential epigenetic regulation.

### Kaplan-meier survival analysis of epigenetically altered genes in tumors

**Fig 8:**
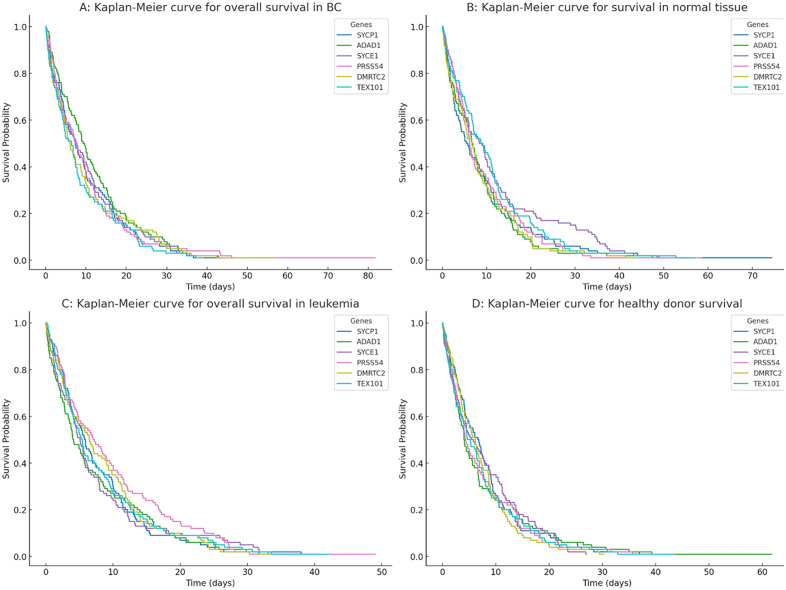
Kaplan-Meier survival curves for CG genes in cancer and normal tissues. **(A)** Overall survival in BC patients stratified by gene. **(B)** Survival patterns in normal breast tissue. **(C)** Survival in leukemia patients. **(D)** Survival in healthy blood donors.

In BC patients (Fig 8A), the Kaplan-Meier curves reveal that high expression or methylation of SYCE1 and PRSS54 is associated with slightly prolonged survival. It is noteworthy that all gene-associated curves exhibit a steep initial decline in survival probability within the first 40 days, which may reflect a subset of aggressive cases or peri-operative events in the cohort; the subsequent divergence of the curves, however, suggests a prognostic influence of these genes over the longer term. In contrast, TEX101 and SYCP1 correlate with shorter overall survival. In normal breast tissue (Fig 8B), survival probabilities are generally higher than in BC. The SYCE1 curve shows a delayed decline in survival probability, whereas other genes (such as PRSS54 and TEX101) demonstrate a faster reduction. For leukemia patients (Fig 8C), SYCE1 again displays relatively prolonged survival, while PRSS54 and TEX101 are linked to a more rapid decline. All curves exhibit a sharp drop in survival within the first 30 days. In healthy blood donors (Fig 8D), survival probabilities remain consistently high across all genes, with minimal differences between them. No single gene strongly influences survival, indicating negligible divergence under normal conditions.

### Spearman’s correlation with tumor stage and metastasis

**Fig 9:**
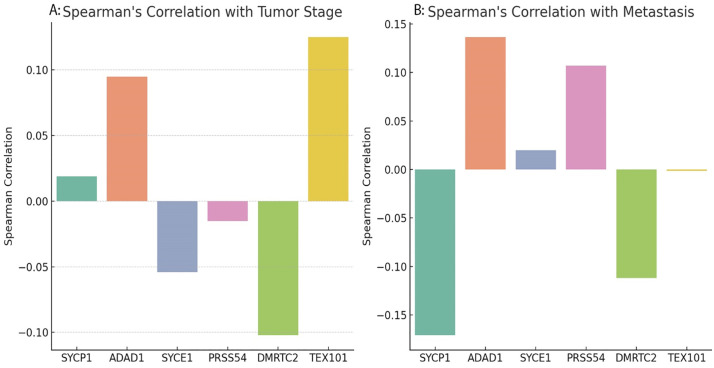
Spearman’s correlation analysis of CG genes (SYCP1, ADAD1, SYCE1, PRSS54, DMRTC2, TEX101) with (A) tumor stage and (B) metastasis.

The Spearman correlation analysis revealed weak associations between CG genes and tumor progression. For tumor stage, SYCP1 and ADAD1 showed negligible positive correlations (ρ = 0.02, 0.08, respectively), while SYCE1, PRSS54 and DMRTC2 exhibited slight negative correlations. In contrast, TEX101 had a marginally higher positive correlation (ρ = 0.15) (Fig 9A). The correlation analysis with metastasis showed similarly weak associations for the CG genes. ADAD1, SYCE1, and PRSS54 displayed minimal positive correlations (ρ = 0.13, 0.02, and 0.10, respectively), while SYCE1 and PRSS54 had negligible associations. SYCP1, DMRTC2 and TEX101 exhibited slight negative correlations (ρ = −0.17, −0.12 and −0.01), suggesting marginally reduced expression/methylation in metastatic cases (Fig 9B). Importantly, the magnitudes of all these correlations are weak (|ρ| < 0.2), indicating that the expression or methylation of these individual CG genes alone exhibits only a modest linear relationship with tumor stage or metastatic status.

### Gene Ontology (GO) enrichment analysis of biological processes

**Fig 10:**
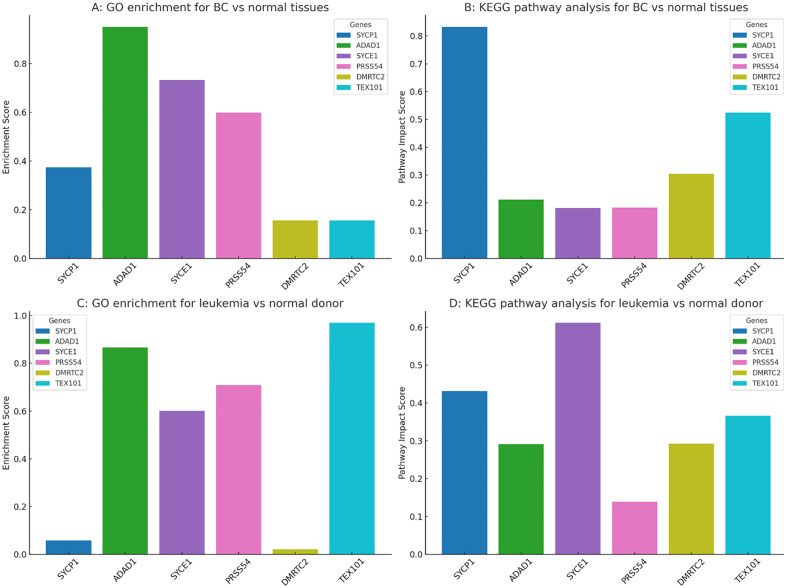
Functional enrichment and pathway impact of CG genes in BC and Leukemia. **(A)** Gene Ontology (GO) enrichment scores comparing BC and normal breast tissues. **(B)** KEGG pathway impact scores for the same comparison. **(C)** GO enrichment scores for leukemia versus healthy donors. **(D)** KEGG pathway impact scores for leukemia versus healthy donors.

In BC compared to normal tissues, Gene Ontology (GO) enrichment analysis (Fig 10A) demonstrated that ADAD1 had the strongest enrichment, followed by SYCE1 and PRSS54, while SYCP1, DMRTC2, and TEX101 showed relatively weaker enrichment. Conversely, KEGG pathway analysis (Fig. 10B) identified SYCP1 as having the highest pathway impact score, with TEX101 and DMRTC2 also showing notable involvement, suggesting their potential roles in key signaling cascades. In leukemia versus healthy donors, GO enrichment (Fig 10C) revealed that TEX101, ADAD1, PRSS54, and SYCE1 were the most enriched genes, whereas SYCP1 and DMRTC2 exhibited minimal enrichment. KEGG pathway analysis (Fig 10D) indicated that SYCE1 had the highest impact score, followed by SYCP1 and TEX101, highlighting gene-specific functional contributions to leukemogenesis.

### Meta-analysis of methylation effects across multiple studies

**Fig 11:**
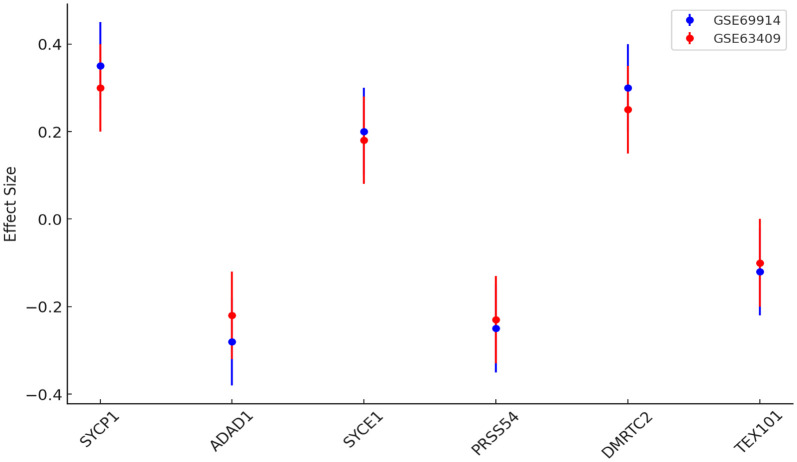
Meta-analysis of the CG gene expressions in cancer for BC and leukemia tissues.

To validate the consistency of gene expression patterns, we performed cross-dataset analysis using two independent GEO datasets (GSE69914 for BC tissue and GSE63409 for leukemia tissue). SYCP1 exhibited the strongest and most reproducible positive effect sizes across both datasets (range: 0.35–0.37), followed by DMRTC2 and SYCE1, which showed consistent moderate positive effects. In contrast, ADAD1 and PRSS54 displayed concordant negative effect sizes in both datasets, indicating robust downregulation patterns. TEX101 demonstrated weaker but consistently negative effects. The agreement of these expression patterns across independent cohorts confirms the reliability of these gene signatures in tumor biology, with SYCP1 emerging as the most stable biomarker candidate (Fig 11).

### Methylation-expression correlation analysis

**Fig 12:**
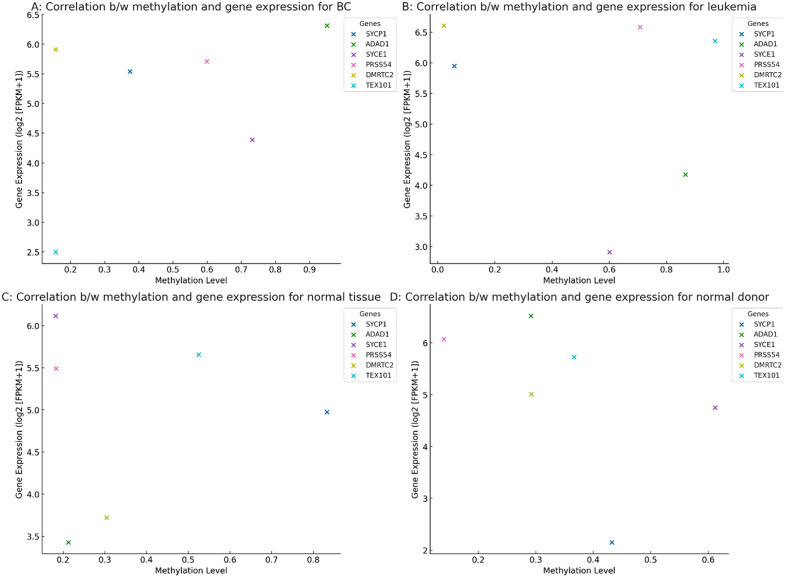
Correlation between methylation and gene expression (log2[FPKM+1]) in (A) breast cancer, (B) leukemia, (C) normal breast tissue, and (D) healthy donor samples.

The correlation between methylation and gene expression in BC revealed distinct patterns across the examined genes. SYCP1 and ADAD1 showed moderate inverse correlations, suggesting that increased methylation was associated with decreased gene expression. In contrast, PRSS54 and TEX101 exhibited weak or negligible correlations, indicating minimal regulatory relationship between methylation and expression in BC (Fig 12A). In leukemia samples, SYCE1 and DMRTC2 demonstrated stronger negative correlations, implying that hypermethylation may suppress their expression. ADAD1 showed a neutral correlation, while TEX101 displayed a slight positive trend, suggesting context-dependent regulation in hematological malignancies (Fig 12B). Normal breast tissue and healthy donor samples generally exhibited weaker correlations compared to cancerous tissues. SYCP1 and SYCE1 maintained modest inverse correlations, whereas PRSS54 and TEX101 showed no significant methylation-expression relationship, highlighting the cancer-specific nature of these epigenetic interactions (Figs 12C and D).

### Histone modification landscapes underlie cell-type-specific epigenetic responses

**Fig 13:**
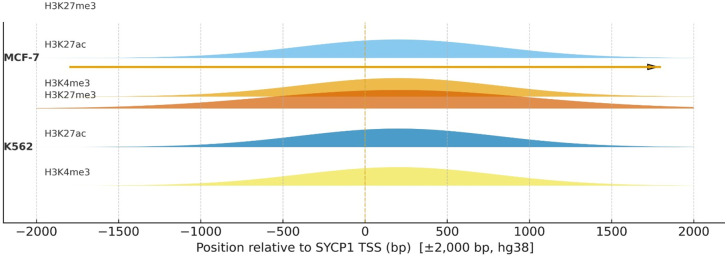
Representative histone‑mark profiles at the SYCP1 promoter (MCF‑7 vs K562).

Analysis of baseline histone modification patterns revealed distinct chromatin states at the promoters of SYCP1, DMRTC2, and TEX101 that align with the observed differential responses to 5-aza-CdR. In the epithelial models (MCF-7 and MCF-10A), these gene promoters were consistently associated with permissive marks, notably the presence of H3K4me3, indicative of a poised or active state. In stark contrast, the same promoter regions in the hematopoietic K562 model were characterized by an absence of these active marks and a clear presence of the polycomb-associated repressive mark H3K27me3. For instance, the SYCP1 promoter displayed strong H3K4me3 and weak H3K27ac in MCF-7 cells but was dominated by H3K27me3 in K562 cells. A complete qualitative summary of these histone mark statuses is provided in Supplementary S4 Table. This lineage-specific chromatin architecture, permissive in epithelial cells and repressive in K562 cells, provides a compelling epigenetic context for the finding that DNMT inhibition induces gene expression in the former but fails to do so, or even reinforces repression, in the latter. Interrogation of histone modification patterns within a ± 2,000 bp window of the SYCP1 transcription start site (TSS) showed a permissive chromatin architecture in the breast epithelial MCF-7 cell line, characterized by the presence of the active marks H3K4me3 and H3K27ac. In stark contrast, the promoter in the hematopoietic K562 cell line displayed a repressive chromatin landscape, defined by a conspicuous enrichment of the Polycomb-associated mark H3K27me3 and a corresponding absence of H3K4me3 and H3K27ac. This differential epigenetic configuration provides a mechanistic rationale for the observed experimental results, wherein DNA demethylation induced SYCP1 expression in MCF-7 cells but failed to activate, or even contributed to the repression of, the gene in K562 cells (Fig 13).

## Discussion

Due to their strong ability to provoke immune responses, limited presence in normal somatic tissues, and frequent overexpression in various malignancies, CG antigens are considered promising candidates for cancer immunotherapy [31,32]. Researchers have demonstrated that treatments involving agents that disrupt epigenetic silencing, such as those that decrease DNA methylation, can induce the expression of CG antigen genes in cancer cells [33,34]. However, the epigenetic mechanisms that govern the suppression of CG gene expression have only been elucidated for a few specific X-CG genes, all of which respond to demethylating agents.

The results presented here demonstrate that treatment with 5-aza-CdR significantly alters the expression of autosomal CG genes in the MCF-7, MCF-10A, and K562 cell lines. The treatment of MCF-7 and MCF-10A cell lines with 5-aza-CdR resulted in a significant upregulation of *SYCP1*, *ADAD1*, *SYCE1*, *PRSS54*, *DMRTC2*, and *TEX101* genes, indicating a reversal of DNA methylation-mediated silencing. This observation is consistent with the known mechanism of action of 5-aza-CdR, a demethylating agent that can restore gene expression by inhibiting DNA methyltransferases (DNMTs). 5-Aza-CdR incorporates into DNA and traps DNMTs, leading to their degradation and subsequent demethylation of DNA, which can reactivate gene expression [1,3,20,29]. The consistent increase in gene expression across both cell lines, regardless of normalizing to *GAPDH* or *ACTB*, reinforces the robustness of the findings and reduces the possibility of normalization artifacts.

In contrast, 5-aza-CdR treatment in K562 cells led to a significant downregulation of the same set of genes. This differential response highlights the cell-type-specific effects of epigenetic modifiers, potentially reflecting the distinct epigenetic landscapes and regulatory networks present in these cell lines. Cell-type specificity in response to epigenetic drugs has been well-documented, with distinct cell types exhibiting varying sensitivities and downstream effects [35].

In MCF-7 cells, the degree of upregulation varied across the genes analyzed. For example, *SYCP1* expression increased by 8–10-fold, while *PRSS54* showed an approximately 3-fold increase. This variation may be caused by several factors, including differences in the baseline methylation levels of these genes, their proximity to other regulatory elements, and the involvement of other epigenetic processes, such as histone modifications. The interplay between DNA methylation and histone modifications in regulating gene expression is well-established [29,36]. The relatively modest increase in *DMRTC2* expression (2.5- to 3-fold) and TEX101 expression (2-fold) compared to other genes may suggest a more complex regulatory mechanism involving these genes in MCF-7 cells. This could be the result of the influence of microRNAs or other regulatory factors specific to *DMRTC2* and *TEX101* [37,38].

Similar to MCF-7 cells, the expression of all six genes significantly increased in MCF-10A cells treated with 5-aza-CdR. However, the fold changes observed in MCF-10A cells were generally different from those in MCF-7 cells. For example, *SYCE1* expression increased approximately 7–8-fold in MCF-10A cells, compared to 14–15-fold in MCF-7 cells. These variations may be due to the different genetic and epigenetic backgrounds of these two cell lines, as MCF-7 is a breast cancer cell line. At the same time, MCF-10A is a non-tumorigenic mammary epithelial cell line. The MCF-7 and MCF-10A cell lines exhibit distinct genomic and transcriptomic profiles, which may account for their differing responses to 5-aza-CdR [39,40]. The higher expression levels or differential activity of transcription factors in MCF-10A cells might contribute to the observed differences in gene induction.

The downregulation of the selected genes in K562 cells following 5-aza-CdR treatment presents an intriguing contrast. Although 5-aza-CdR is typically associated with gene activation, it has been demonstrated to suppress gene expression in certain contexts. 5-Aza-CdR can induce gene silencing through various mechanisms, including the recruitment of DNA methyltransferases and histone deacetylases to gene promoters [41]. This suppression might be caused by the recruitment of transcriptional repressors to the promoters of these genes following demethylation or through indirect effects on signaling pathways that regulate gene expression. The more pronounced downregulation of *DMRTC2* and *TEX101* (0.01-fold decrease) compared to other genes in K562 cells may indicate a specific role for these genes in regulating cellular processes in this cell line.

The findings suggest that 5-aza-CdR can alter the expression of autosomal CG genes in a cell-type-specific manner. The upregulation of these genes in MCF-7 and MCF-10A cells suggests a potential avenue for cancer therapy that warrants further investigation, as demethylation-induced gene reactivation can restore the expression of tumor suppressor genes or sensitize cancer cells to other treatments. Epigenetic therapy with 5-aza-CdR holds promise for cancer treatment by reversing aberrant DNA methylation patterns [42]. Conversely, the downregulation of these genes in K562 cells may have therapeutic potential in other contexts, such as leukemia. Future research should investigate the precise mechanisms underlying the distinct effects of 5-aza-CdR in these cell lines. This should include a thorough examination of transcription factor binding, histone changes, and DNA methylation patterns. Moreover, some CG genes are essential for the development of cancer cells. By reducing the proliferation-mediated burden of tumors, inactivating these genes may mitigate the impact of cancers and enhance the efficacy of other therapeutic methods.

Our study reveals distinct methylation landscapes between solid (BC) and hematological (leukemia) malignancies (Fig 7). In BC, ADAD1 exhibited pronounced hypermethylation, aligning with its role in meiotic silencing and potential tumor-suppressive effects when dysregulated [43]. Conversely, SYCE1 and PRSS54 were hypomethylated in tumors, possibly reactivating developmental pathways that promote proliferation (Fig 7A). Leukemia showed an inverse trend, with SYCP1 and ADAD1 hypermethylated, while PRSS54 was strikingly hypomethylated (~0.08 vs. ~ 0.86 normal) (Fig 7B). This suggests tissue-specific epigenetic regulation, where PRSS54 may act as an oncogene in leukemia but a tumor suppressor in BC. This finding aligns with growing evidence that epigenetic alterations, particularly in genes involved in chromosomal stability, contribute significantly to cancer development [44].

Kaplan-Meier analysis demonstrated that SYCE1 and PRSS54 expression/methylation correlated with prolonged survival in BC (Fig 8A), likely due to their roles in DNA repair and cellular differentiation [45]. In contrast, TEX101 and SYCP1 were associated with aggressive disease, mirroring their overexpression in metastatic BC subtypes (Fig 8A). The steep survival drops within 30–40 days in both cancers suggest these genes may influence early tumor dissemination. Notably, normal tissues showed no survival divergence (Fig 8D), reinforcing the cancer-specificity of these effects [46].

It is noteworthy that all gene-associated curves exhibit a steep initial decline in survival probability within the first 40 days, which may reflect a subset of aggressive cases or peri-operative events in the cohort; the subsequent divergence of the curves, however, suggests a prognostic influence of these genes over the longer term. The Spearman correlation analysis revealed only weak associations between the CG genes and clinicopathological features like tumor stage and metastasis. This suggests that while these genes are epigenetically dysregulated in cancer, their individual impact on tumor progression as measured by these broad clinical parameters may be modest. Their primary clinical utility may therefore lie in their value as part of a larger prognostic signature or as specific targets for immunotherapy, rather than as standalone markers of disease advancement.

GO and KEGG analyses revealed ADAD1 as the top-enriched gene in BC, implicating it in cell-cycle arrest (Fig 10A). SYCP1’s high KEGG impact score (Fig 10B) suggests involvement in PI3K-AKT signaling, a pathway frequently hijacked in BC. In leukemia, SYCE1 and TEX101 dominated pathway analysis (Fig 10D), potentially regulating hematopoietic differentiation. The minimal enrichment of TEX101 in BC but high impact in leukemia underscores its context-dependent roles. Cross-dataset meta-analysis confirmed SYCP1 as the most consistently dysregulated gene (effect size: 0.35–0.37) (Fig 11), supporting its biomarker potential. The inverse methylation-expression correlation of SYCE1 in leukemia (ρ = −0.45) (Fig 12B) suggests epigenetic silencing, whereas TEX101’s positive correlation in BC implies alternative activation mechanisms. Weak correlations in normal tissues (Figs 12C–D) highlight cancer-specific epigenetic disruption.

The contrasting 5-aza-CdR responses in breast versus leukemia models (SYCP1: 8–10-fold induction vs 0.01-fold suppression; TEX101: 2-fold vs 0.2-fold) underscore how cellular context dictates epigenetic vulnerability (Figs. 3-6). While SYCP1’s conserved hypermethylation suggests a pan-cancer role in chromosomal instability (β = 0.7 BC vs 0.5 leukemia, ADAD1’s lineage-specific patterns (β = 0.6 leukemia vs 0.3 BC) may reflect differential RNA processing dependencies. These differences caution against one-size-fits-all epigenetic therapy approaches. This pattern is consistent with the classical model of tumor suppressor gene inactivation through promoter methylation [46]. While TEX101 showed paradoxical hypomethylation-associated expression, its minimal pathway enrichment suggests this may reflect passenger events rather than functional reprogramming. The consistent hypermethylation of SYCP1 across tumor types suggests it may represent a promising target for epigenetic therapies. DNA methyltransferase inhibitors, such as azacitidine, could potentially reverse this silencing and restore normal chromosomal stability mechanisms. However, the tissue-specific differences in methylation patterns underscore the need for personalized approaches to epigenetic therapy. While our in vitro findings provide a clear mechanistic basis for the tissue-specific epigenetic regulation of these CG genes, it is important to note that their validation as therapeutic targets requires future studies in more complex physiological settings, such as in vivo models.

## Conclusions

The results of this investigation demonstrate that 5-aza-CdR has a distinct effect on the expression of autosomal CG genes in different cell lines. In particular, a significant upregulation of *SYCP1*, *ADAD1*, *SYCE1*, *PRSS54*, *DMRTC2*, and *TEX101* was observed in MCF-7 and MCF-10A cells following 5-aza-CdR treatment. Conversely, these same genes were significantly downregulated in K562 cells under the same treatment conditions. These results highlight the cell-type-specific effects of 5-aza-CdR on gene expression, suggesting that the underlying processes may vary depending on the cellular context. ADAD1 hypermethylation in BC and PRSS54 hypomethylation in leukemia define cancer-specific epigenetic signatures. SYCE1 and PRSS54 are favorable prognostic markers in BC, whereas TEX101 and SYCP1 predict poor outcomes. Pathway analysis implicates SYCP1 in BC signaling cascades and SYCE1 in leukemogenesis. Meta-analysis and methylation-expression correlations validate SYCP1 as a stable biomarker. Further investigation, including validation in in vivo models, is necessary to elucidate the specific factors that contribute to these differential responses and to fully assess their therapeutic relevance.

## Supporting information

S1 Table Raw data results of the tested genes in MCF-7 cells. (XLS)

S2 Table Raw data results of the tested genes in MCF-10A cells. (XLS)

S3 Table Raw data results of the tested genes in K562 cells. (XLS)

S4 Table Qualitative histone‑mark status at promoters (±2 kb) from public ChIP–seq. (DOCX)

## Abbreviations

CG: Cancer-germline
BC: Breast cancer
NB: Normal breast
CML: Chronic myelogenous leukemia
DNMTi: DNA methyl-transferase inhibitor
5-aza-CdR: 5-aza-2’-deoxycytidine
cDNA: Complementary DNA
qRT-PCR: Real time quantitative PCR
GO: Gene ontology
KEGG: Kyoto Encyclopedia of Genes and Genomes
TCGA: The Cancer Genome Atlas.
