# Supplementary material for: Contrasting effects of DNA demethylation on cancer-germline gene expression in breast cancer and leukemia cells
Source: PLoS One. 2025 Dec 18;20(12):e0339460. doi: 10.1371/journal.pone.0339460 (PMC12714251; doi:10.1371/journal.pone.0339460)
Supplement: S4 Table — (DOCX) [file pone.0339460.s004.docx]

**S4 Table. Qualitative histone‑mark status at promoters (±2 kb) from public ChIP–seq**

| **Gene** | **Cell model** | **H3K4me3** | **H3K27ac** | **H3K27me3** | **Data source(s)** |
| --- | --- | --- | --- | --- | --- |
| SYCP1 | MCF‑7 | present | present/weak | low | ENCODE ENCSR000DWJ; ENCSR000EWR; ENCSR000EWP |
| SYCP1 | K562 | low/absent | absent | present | ENCODE ENCSR000AKU; ENCSR000EWB |
| SYCP1 | MCF‑10A | present | present/weak | low | GEO GSM2258705; Roadmap |
| DMRTC2 | MCF‑7 | present | present/weak | low | ENCODE |
| DMRTC2 | K562 | low/absent | absent | present | ENCODE |
| DMRTC2 | MCF‑10A | present | present/weak | low | Roadmap/GEO |
| TEX101 | MCF‑7 | low/absent | absent/weak | variable/low | ENCODE |
| TEX101 | K562 | absent | absent | present | ENCODE |
| TEX101 | MCF‑10A | low/absent | absent/weak | variable/low | Roadmap/GEO |
